# On-site clinical mentoring as a maternal and new-born care quality improvement method: evidence from a nurse cohort study in Nepal

**DOI:** 10.1186/s12912-019-0396-1

**Published:** 2020-01-08

**Authors:** Sophie Goyet, Swaraj Rajbhandari, Valerie Broch Alvarez, Aida Bayou, Sirjana Khanal, Tara Nath Pokhrel

**Affiliations:** 1 7 passage du Clair Matin, 74940 Annecy le Vieux, France; 2 Management 4 health -GmbH, Hebelstrasse 11, 60318 Frankfurt, Germany; 3 Deutsche Gesellschaft fur International Zusammernarbeit (GIZ), Kathmandu, Sanepa, Nepal; 4 Family Health Division, Ministry of Health and Population, Government of Nepal, Ministry of Health and Population, Ramshaha Path, GPO Box: 7830, Kathmandu, 44600 Nepal

**Keywords:** Nepal, Asia, Human resources for health, Maternal and new-born care, Mentoring, Emergency obstetrical and new-born care

## Abstract

**Background:**

We describe an on-site clinical mentoring program aimed at improving emergency obstetrical and new-born care (EmONC) in Nepal and assess its effectiveness on nurses’ knowledge and skills. In Nepal, both the maternal mortality ratio (MMR, 239/100,000 live births) and the neonatal mortality rate (NMR, 21/1000 live births) were among the highest in the world in 2016, despite impressive progress over recent decades considering the challenging environment.

**Methods:**

From September 2016 to April 2018, three experienced nurses conducted repeated mentoring visits in 61 comprehensive or basic EmONC centers and birthing centers located in 4 provinces of Nepal. Using updated national training manuals and teaching aids, these clinical mentors assessed and taught 12 core EmONC clinical skills to their nurse-mentees. Clinical mentors worked with management mentors whose goal was to improve the nurses’ working environment. We assessed whether the cohort of nurse-mentees performed better as a group and individually performed better at the end of the program than at baseline using relevant tests (chi-square test, Wilcoxon matched-pairs signed-rank test, and Kruskal-Wallis equality-of-population rank test).

**Results:**

In total, 308 nurses were assessed, including 96 (31.2%), 77 (25.0%) and 135 (43.8%) who participated in all three, two or only one mentoring session, respectively. In total, 225 (73.0%) worked as auxiliary nurse-midwives (ANMs), while 69 (22.4%) worked as nurses. One hundred and ninety five (63.3%) were trained as skilled birth attendants, of which 45 (23.1%) were nurses, 141 (72.3%) were auxiliaries and 9 (4.6%) had other positions. The proportion of ANMs and nurse-mentees who obtained a knowledge assessment score ≥ 85% increased from 57.8 to 86.1% (*p* <  0.001). Clinical assessment scores increased significantly for each participant, and therefore for the group. SBA-trained mentees had better knowledge of maternal and new-born care and were better able to perform the 12 core clinical skills throughout the program.

**Conclusions:**

Our study suggests that on-site clinical mentoring of nurses coupled with health facility management mentoring can improve nurses’ clinical competences in and performance of maternity and new-born care. Assessing evidence of impact on patient safety would be the next stage in evaluating this promising intervention.

## Introduction

Nepal is a landlocked, low-income country with geographical conditions that make it one of the most disaster-prone countries worldwide (earthquakes, river floods and landslides) [1]. Nepal also endured years of civil war, from 1996 to 2006 [2, 3]. However, this country has made impressive progress over recent decades in child survival and maternal health, despite these major challenges [4]. The maternal mortality ratio (MMR) decreased between 1996 and 2016 from 539 to 239 per 100,000 live births [4]. The neonatal mortality rate (NMR), estimated at 50 per 1000 live births in 1996, was still among the highest in the world in 2016 (21/1000) [4, 5].

The observed progress can be attributed to government efforts, which, after the civil war in the 1990s, demonstrated a consistent policy focus on maternal and child health, with sustained financial commitment and funding for maternal health through the national program ‘Aama Surkshya’. This program was implemented by the Family Health Division (FHD) of the Ministry of Health and Population (MoHP) in 2005 and offers cash to women who deliver at a health facility and to health care providers who attend the deliveries [6], which contributed to the increase in institutional deliveries (from 18% in 2006 to 57% in 2017 [4, 7]). Improving the quality of care became a focus of national health strategies in 2016 [8] and remains a current goal in the context of Nepal’s commitment to universal health coverage [9]. Substantial progress is still needed to achieve the country’s sustainable development goals for 2030 (a MMR of 70/100,000 and a NMR of 12/1000) [4, 5].

Mentorship of health workers was introduced in Nepal by the FHD/MoHP in 2014 with the support of the Deutsche Gesellschaft fur International Zusammernarbeit (GIZ), which channels the German technical cooperation, and its bilateral Nepal-Germany program aimed at improving maternal and new-born care (MNC). The decision to implement this program was based on the need to follow-up on a MNC provider training program run by the Nick Simons Institute and the FHD of the MoHP, as well as on preceding experience in mentoring to expand abortion care [10]. The initial phase of this program lasted from July 2014 to July 2016 and was initiated due to the stagnation of the MMR and NMR and the poor performance of staff working in maternity settings. Its core intervention was the mentoring of emergency obstetrical and new-born care (EmONC) health staff by national senior peers through a bundled approach combining mentorship of both clinical and management teams working in EmONC facilities. This initial phase was shortened due to a strategy reorientation after the major earthquake in April 2015, which killed 8897 people and destroyed 83.5% of public health facilities in the most affected area [1].

A second phase of this mentoring program was implemented in 2016 at the request of the MoHP. This phase included some districts affected by the earthquake, expanded the mentorship to the staff working in birthing centers and promoted the use of tools developed by the National Health Training Center for skilled birth attendants (SBAs). The objectives of this second phase were to i) improve the clinical capacity of MNC service providers, ii) improve the quality of management in health facilities, and iii) support referral networks. Mentoring was considered a comprehensive teaching and learning process in which senior health workers, who served as the mentors, provided personalized support to juniors, who were the mentees, based on the mentees’ needs, to strengthen their ability to provide quality health care [11].

Different mentoring models have been used in diverse country contexts for supporting clinical staff, ranging from field-based teams of mainly non-physician health workers to highly skilled mentors (specialists in obstetrics/gynaecology and paediatrics) and nurse mentors in India and Rwanda [11–13]. However, the effectiveness of mentoring programs for health staff is not yet well documented in the scientific literature. Available studies mostly focus on institutional mentoring for medical or nursing students [14–17]. Several studies have examined mentors’ and mentees’ perceptions of their experiences, challenges and needs [18, 19]. A recent study on the effectiveness of on-site mentoring in Indian maternity clinics examined the impact of mentoring on nurses’ knowledge and clinics’ readiness to provide services but did not consider the effectiveness of mentoring on provider practices [20]. A Cochrane review noted the need for further evaluations in different settings and contexts [21].

The objective of this paper is to describe a mentoring program aimed at improving emergency MNC in Nepal and to assess its effectiveness on nurses’ knowledge and skills.

## Methods

We prospectively performed repeated cross-sectional assessments of the knowledge and clinical skills of a cohort of nurses working in comprehensive or basic EmONC centers (CEONCs and BEONCs) and birthing centers in Nepal. These nurses work either as ‘staff nurses’ (they should have received at least a 3-year pre-service nursing training) or as auxiliary nurse-midwives (ANMs) (their pre-service training lasts only 18 months). Some ANMs and staff nurses were certified as SBAs after completion of a 2-month SBA training. Both types of nurses, SBA certified or not, should be able to attend to women in labour, managing both normal and complicated deliveries (before transfer to a higher facility for appropriate care whenever it is possible). In remote birthing centers, ANMs often work alone.

The mentoring program lasted from September 2016 to April 2018 in seven CEONCs, 19 BEONCs and 35 birthing centers in seven/72 districts of Province no. 7, 5, 6 and 3 in Nepal. These health facilities represent, respectively, 6.7, 11.9 and 2.0% of all CEONCs, BEONCs and birthing centers existing in 2015 [8]. Districts and health facilities were purposely selected by the MoHP to represent the diversity of Nepal’s geographical and economic environment. Some of these districts belong to the poorest and most remote areas in Nepal. Three of them were also particularly affected by the 2015 Nepal earthquake. All CEONC and BEONC centers in these districts were included in the program, as well as the birthing centers having the highest patient flow or the highest number of pregnancies.

### Clinical mentoring organization

The clinical mentors organized three mentoring sessions (each lasting three to four days) in each targeted health facility between December 2016 and March 2018. A total of 12 MNC topics were covered (some of them introduced during the second mentoring session only): completing a normal delivery and resuscitating a new-born, plotting and interpreting a partograph, managing shock due to a post-partum haemorrhage, safely referring a woman or a new-born to a higher-level health facility, performing a vacuum delivery, performing manual aspiration, promoting kangaroo mother care, managing an eclampsia case, decontaminating used medical equipment and using sterile gloves.

**Fig. 1:**
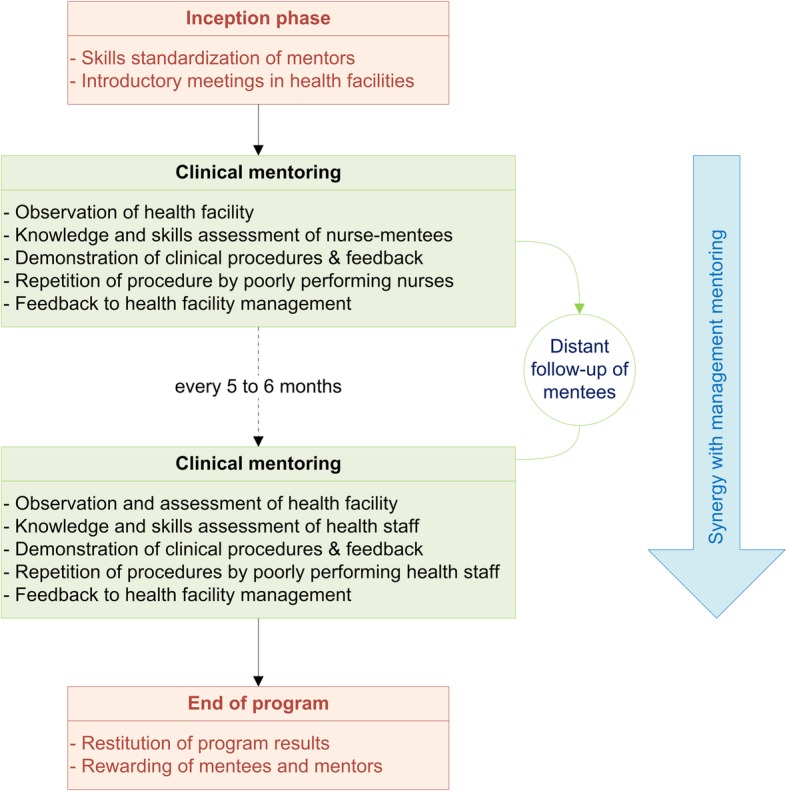
Organization of the clinical mentoring, Nepal, 2016–2018

In accordance with the standards-based management and recognition approach developed by Jhpiego, which has been used since 1997 and has been implemented in more than 30 countries [22], the mentoring sessions were organized as follows; Mentoring sessions began with an introductory meeting and a visit of the health facility (Fig. 1). Then, each nurse-mentee was individually assessed regarding her knowledge in MNC, as well as her ability to perform 12 clinical skills (after they had been trained in these skills), using quality improvement (QI) tools approved by the MoHP. Feedback was then given to each mentee before the mentor demonstrated the correct steps using manikins. Poorly performing mentees were invited to repeat the procedure. Where possible, mentoring was reinforced through engagement with real patients. Mentoring visits concluded with a meeting with the mentees, the health facility management team and the respective district health authorities whenever possible. A joint action plan was then prepared, developing actions to be taken to close the observed gaps until the next mentoring session, which occurred five to six months later. Mentorship follow-up was offered by phone when necessary.

The 14 clinical mentors were nursing graduates with extensive maternity and public health and training experience, selected with the help of the Ministry of Health based on technical criteria and their communications skills and experience; four of them were SBA trainers. They were trained on the mentoring approach and QI tools in October 2016 and were supervised by the program team.

### Complementary management mentoring

Clinical mentors worked in pairs with management mentors who supported health facility management teams. In a given health facility, clinical and management mentors jointly conducted mentoring sessions. The management mentors worked with the health facility heads and management committees and covered eight quality care domains: health facility management, infrastructure, patient dignity, staffing, supplies and equipment, drugs, and infection prevention (Additional file 1). Concomitant mentoring activities were also organized regarding advanced MNC practices with the help of specialized mentors for CEONC and neonatal care staff.

### Knowledge and skills assessments

Standardized assessments using multiple choice questions and an objective structured clinical examination were used to measure the changes in knowledge and clinical skills during the program.

The nurse-mentees’ knowledge was assessed at the beginning of each mentoring session using an auto-administered questionnaire in the Nepali language extracted from the national SBA training manual [23]. It consisted of 26 questions on the following topics: antenatal care, partograph, normal delivery, vacuum delivery, complicated procedures, new-born resuscitation, eclampsia, and infection prevention (detailed list shown in Additional file 2). Knowledge assessment scores were calculated as the proportion of correct answers to the 26 questions.

The mentees were also assessed before each mentoring session for 12 core clinical skills using the QI tools extracted from the same SBA training manual. Each QI tool consisted of a set of questions to answer or procedures to demonstrate grouped into ‘standards’ with verification criteria. The expected answers, procedure steps and verification criteria were detailed in checklists attached to the QI tools. For each assessed skill, a skill score was computed as the number of standards fully and correctly performed over the total number of standards; these results were presented as a percentage. Managing an eclampsia crisis was not assessed among non-SBAs during the first mentoring session. The referral procedure and condom tamponade were introduced during the second mentoring session only. Manual vacuum aspiration and vacuum delivery were assessed in only CEONCs and some BEONCs where the necessary equipment was available. We called the ‘overall clinical score’ the mean of results obtained for the 12 clinical skills assessed, expressed as percentages.

### Analyses

The clinical mentors recorded the mentees’ assessment scores using an Excel sheet or the KoBo Toolbox [24]. Data sets were anonymized using a unique identifier for each mentee. Characteristics of the mentees are presented as medians and interquartile ranges (IQRs) for continuous data and frequencies and percentages for categorical data.

We assessed mentees’ knowledge and skills at baseline and determined whether they performed better according to their nursing education, their SBA status, their function and place of employment using the chi-square test. Mentees who were not engaged in daily clinical practice (i.e., not working as ANMs or nurses) were removed from the analyses. The National Health Training Center and the program team defined good knowledge and performance scores as scores equal to or above 85%.

We then assessed whether the mentees performed better as a group at the end of the program than at baseline. We therefore compared the proportion of ANMs and nurses with scores ≥85% between the first and last mentoring sessions using the chi-square test.

We completed the analysis by assessing the change in mentees’ assessment scores from baseline to the end of the program according to their SBA status using the Kruskal-Wallis equality-of-population rank test. The mentees who became SBAs during the program were removed from the analysis comparing SBAs with non-SBAs.

Finally, since not all mentees attended all mentoring sessions, we also assessed whether they individually performed better at the end of the program using the Wilcoxon matched-pairs signed-rank test. In this test, each mentee was matched with herself at baseline and at the end of the program, and her first assessment was used as the point of reference for comparisons.

All analyses were performed using Stata 13 (Stat Corp., College Station, TX, USA) software. Statistical significance was defined at a 5% threshold (*p* <  0.05).

### Ethical considerations

This article does not report on a health research involving human subject as stated by the Nepal Health Research council (file:///C:/Users/Acer/Downloads/ERB_Guideline_2019-final-_29-Sep-1.pdf). It is a review of an educational program jointly implemented with the MoHP of Nepal. Delivery and evaluation of this educational program were approved by and implemented with the MoHP. All nurses involved in this education program provided their oral consent to participate in the program, and were allowed to drop out anytime. Their consent was recorded in the anonymized program follow-up database. Knowledge and skills assessments were considered part of the learning experience. Databases that recorded assessment results were anonymized, with access restricted to data analysts and program managers.

## Results

The clinical mentoring program reached a total of 308 nursing staff. Of them, 196 benefitted from the first mentoring session, 197 from the second session and 184 from the last one. A total of 96 mentees (31.2%) participated in all three mentoring sessions, 77 (25.0%) received two mentoring sessions, and 135 (43.8%) were reached only once (Table 1).

**Table 1:** Characteristics of the nurses mentored in 35 birthing centers, 19 BEONCs, and 7 CEONCs in Nepal from 2016 to 2018

	Birthing centers		BEONC centers^a^		CEONC centers^a^		All	
	*n*	*%*	*n*	*%*	*n*	*%*	*n*	*%*
No. of nurse-mentees	100	32.5	92	29.9	116	37.7	308	100.0
Female gender	100	100.0	92	100.0	116	100.0	308	100.0
Age (in years): median [IQR] ^b^	29	[24–35]	32	[25–39]	26	[24–35]	29	[24–38]
Level of nursing education								
3-year auxiliary nurse-midwife course	79	79.0	53	57.6	50	43.1	182	59.1
3-year Proficiency Certificate Level	14	14.0	19	20.6	40	34.5	73	23.7
Bachelor’s degree in nursing	4	4.0	17	18.5	24	20.7	45	14.6
Missing information	3	3.0	3	3.3	2	1.7	8	2.6
Skilled birth attendant (SBA) status								
not a SBA ^c^	42	42.0	29	31.5	42	36.2	113	36.7
SBA	58	58.0	63	68.5	74	63.8	195	63.3
Status at first contact ^d^								
Auxiliary nurse-midwife	94	94.0	67	72.8	64	55.2	225	73.0
Nurse	3	3.0	22	23.9	44	37.9	69	22.4
Other	0	0.0	0	0.0	6	5.2	6	1.9
Missing information	3	3.0	3	3.3	2	1.7	8	2.6
Experience in years: median [IQR] ^b^	6	[3–10]	10	[5–18]	5	[2–13]	7	[3–14]
Program intensity								
3 mentoring sessions	42	42.0	33	35.9	21	18.1	96	31.2
2 mentoring sessions	26	26.0	25	27.2	26	22.4	77	25.0
1 mentoring session	32	32.0	34	37.0	69	59.5	135	43.8

^a^CEONC: comprehensive emergency obstetrical and new-born care; BEONC: basic emergency obstetrical and new-born care

^b^Available for 300 mentees

^c^Including 4 staff who specialized in SBA during the course of the program

^d^ANMs should be support staff nurses, but they often work alone

All 308 mentees were female (Table 1). Their median age was 29 years (IQR: 24–38), and they had a median work experience of 7 years (IQR: 3–14). More than half of them (*n* = 182, 59.1%) had undergone an 18-month pre-service ANM training course. Seventy-three mentees (23.3%) had a nursing certificate obtained after a 3-year nursing course, and 45 (14.6%) had a bachelor’s degree in nursing. A total of 195 mentees (63.3%) were trained as SBAs, including 45 nurses (23.1%), 141 ANMs (3.1%), and 9 with other or unknown positions (4.6%). Of all the mentees, 225 (73.0%) worked as ANMs and 69 (22.4%) worked as nurses.

### Mentees’ assessments at baseline

During the first mentoring session, 185 ANMs and nurses completed the initial knowledge assessment, with 107 (57.8%) of them correctly answering 85% or more of the 26 questions (Table 2). SBAs had a higher level of knowledge than non-SBAs (64.8% of SBAs obtained a score ≥ 85%, vs 34.9% among non-SBAs, *p* = 0.001). Staff working in CEONCs also had better knowledge than staff in BEONCs and birthing centers (72.5, 67.7 and 38.9%, respectively, *p* < 0.001). We did not find evidence that staff with higher nursing education performed better in the knowledge assessment than less educated staff or that nurses had better knowledge than ANMs.

**Table 2:** Knowledge and overall skill assessment results at baseline and the end of the program in Nepal from 2016 to 2018

	Baseline			End of Program			Progress		
	Tested	Obtained a score ≥ 85%	Chi-square Pearson	Tested	Obtained a score ≥ 85%	Chi-square Pearson	Change		*Chi-square Pearson*
	N	n (%)	*P*	N	n (%)	*P*			*P*
Knowledge assessment: all	185	107 (57.8)	–	180	180 (86.1)	–	+	28.3	< 0.001
*Education*									
18-month ANM course	119	63 (52.9)	*NS*	119	119 (83.2)	*NS*	+	30.3	*< 0.001*
3-year PCL	44	29 (65.9)		39	39 (87.2)		+	21.3	*0.02*
Bachelor in nursing	22	15 (68.2)		22	22 (100.0)		+	31.8	*0.004*
*SBA status*									
Not trained	43	15 (34.9)	*0.001*	62	44 (71.0)	*< 0.001*	+	36.1	*< 0.001*
SBA-trained	142	92 (64.8)		118	111 (94.1)		+	29.3	*< 0.001*
*Function*									
ANMs	149	83 (55.7)	*NS*	142	120 (84.5)	*NS*	+	28.8	*< 0.001*
Staff nurses	36	24 (66.7)		38	35 (92.1)		+	25.4	*0.007*
*Location*									
Birthing centers	72	28 (38.9)	*< 0.001*	66	60 (90.9)	*NS*	+	52.0	*< 0.001*
BEONCs	62	42 (67.7)		58	58 (89.7)		+	22.0	*0.004*
CEONCs	51	37 (72.5)		56	43 (77.0)		+	4.5	*NS*
Clinical skills assessment: all	186	14.5	***–***	180	139 (77.2)	–	+	62.7	*< 0.001*
*Education*									
18-month ANM course	120	11.7	*NS*	119	94 (79)	*NS*	+	67.3	*< 0.001*
3-year PCL	44	20.4		39	29 (74.4)		+	54.0	*< 0.001*
Bachelor in nursing	22	18.2		22	16 (72.7)		+	54.5	*< 0.001*
*SBA status*									
Not trained	43	4.6	*0.03*	62	33 (53.2)	*< 0.001*	+	48.6	*< 0.001*
SBA-trained	143	17.5		118	106 (89.8)		+	72.3	*< 0.001*
*Function*									
ANMs	150	11.3	*0.01*	142	112 (78.9)	*NS*	+	67.6	*< 0.001*
Staff nurses	36	27.8		38	27 (71.0)		+	43.2	*< 0.001*
*Posting*									
Birthing centers	73	4.1	*< 0.001*	66	56 (84.8)	*0.04*	+	80.7	*< 0.001*
BEONCs	62	9.7		58	46 (79.3)		+	69.6	*< 0.001*
CEONCs	51	35.3		56	37 (66.1)		+	30.8	*0.001*

Only mentees working as auxiliary nurse-midwives or nurses who had baseline and end of program assessments for the skills assessed were included in the assessment

Baseline scores were measured during the first mentoring session (Sept to Nov 2016)

End scores were measured during the last mentoring session (Jan to Mar 2018)

*ANM* auxiliary nurse-midwife, *PCL* Proficiency Certificate Level, *CEONC* comprehensive emergency obstetric and new-born care center, *BEONC* basic emergency obstetric and new-born care center

Of the 186 ANMs and nurses assessed for at least one clinical skill at baseline, 27 (14.5%) obtained a good overall clinical score (Table 2). Nurses performed better than ANMs (27.8% of the nurses obtained a good score vs 11.3% of ANMs, *p* = 0.01); SBAs performed better than non-SBAs (17.5% vs 4.6%, respectively, *p* = 0.03), as well as staff working in CEONCs (35.3% vs 9.7% in BEONCs and 4.1% in birthing centers, *p* < 0.001). We did not find evidence of any association between the level of nursing education and clinical performance at baseline.

The mentees’ clinical performance varied widely at baseline, from 91.5% of them knowing how to put on sterile gloves correctly to 13.4% of them being able to manage a case of shock due to a post-partum haemorrhage (Table 3). Less than one-fourth of the mentees were able to correctly manage an eclampsia case (13.9%), conduct a normal delivery following national standards (18.2%) and interpret a partograph (20.2%). At baseline, our data did not show any difference in performance between the nurses and the ANMs, except for ‘managing an eclampsia case’ (26.7% of nurses obtained a good score vs 10.7% of ANMs, *p* = 0.02; Table 3). The skills performed better by SBAs than non-SBAs at baseline were the following: new-born resuscitation, interpretation of a partograph and decontamination of instruments (data not shown).

**Table 3:** Results of skills assessments measured at baseline and at the end of the program in Nepal, 2016–2018

	At baseline ^a^			At end ^b^			Comparison baseline - end	
	*Tested*	*Obtained a score ≥ 85%*		*Tested*	*Obtained a score ≥ 85%*		*Change*	*Chi-square Pearson*
	N	%	*P*	N	%	*P*		*P*
Managing an eclampsia case: all	151	13.9		171	70.8		+ 56.9	*< 0.001*
Auxiliary nurse-midwives only	121	10.7	*0.02*	134	73.1	*NS*	+ 62.4	*< 0.001*
Nurses only	30	26.7		37	62.2		+ 35.5	*0.004*
Managing shock due to PPH	149	13.4		170	70.6		+ 57.2	*< 0.001*
Auxiliary nurse-midwives	118	11.9	*NS*	133	71.4	*NS*	+ 59.5	*< 0.001*
Nurses	31	19.3		37	67.6		+ 48.3	*< 0.001*
Resuscitating a new-born	169	26.0		179	82.7		+ 56.7	*< 0.001*
Auxiliary nurse-midwives	135	23.0	*NS*	141	85.1	*NS*	+ 62.1	*< 0.001*
Nurses	34	38.2		38	73.7		+ 35.5	*0.002*
Completing a normal delivery	170	18.2		179	73.2		+ 55.0	*< 0.001*
Auxiliary nurse-midwives	134	15.7	*NS*	141	75.2	*NS*	+ 59.5	*< 0.001*
Nurses	36	27.8		38	65.8		+ 38.0	*0.001*
Safely referring a woman or a new-born	131	26.7		169	76.9		+ 50.2	*< 0.001*
Auxiliary nurse-midwives	105	25.7	*NS*	134	79.1	*NS*	+ 53.4	*< 0.001*
Nurses	26	30.8		35	68.6		+ 37.8	*0.003*
Interpreting a partograph	183	20.2		180	68.9		+ 48.7	*< 0.001*
Auxiliary nurse-midwives	147	21.1	*NS*	142	66.9	*NS*	+ 45.8	*< 0.001*
Nurses	36	16.7		38	76.3		+ 59.6	*< 0.001*
Promoting kangaroo mother care	108	28.7		175	78.9		+ 50.2	*< 0.001*
Auxiliary nurse-midwives	91	27.5	*NS*	138	79.7	*NS*	+ 52.2	*< 0.001*
Nurses	17	35.3		37	75.7		+ 40.4	*0.004*
Performing condom tamponade ^c^	133	61.6		150	85.3		+ 23.7	*< 0.001*
Auxiliary nurse-midwives	102	59.8	*NS*	117	85.5	*NS*	+ 25.7	*< 0.001*
Nurses	31	67.7		33	84.8		+ 17.1	*NS*
Decontaminating medical equipment	97	78.3		176	93.7		+ 15.4	*< 0.001*
Auxiliary nurse-midwives	84	76.2	*NS*	139	94.7	*NS*	+ 18.5	*< 0.001*
Nurses	13	92.3		37	89.2		- 3.1	*NS*
Using sterile gloves: all mentees	153	91.5		176	98.9		+ 7.4	*0.001*
Auxiliary nurse-midwives	119	89.9	*NS*	139	98.6	*NS*	+ 8.7	*0.002*
Nurses	34	97.1		37	100.0		+ 2.9	*NS*
Performing manual aspiration ^d^	29	48.3		63	76.2		+ 27.9	*0.008*
Auxiliary nurse-midwives	19	52.6	*NS*	44	70.4	*NS*	+ 17.8	*NS*
Nurses	10	40.0		19	89.5		+ 49.5	*0.005*
Performing a vacuum delivery ^d^	72	48.6		67	85.1		+ 36.5	*< 0.001*
Auxiliary nurse-midwives	50	42.0	*NS*	48	83.3	*NS*	+ 41.3	*< 0.001*
Nurses	22	63.6		19	89.5		+ 25.9	*NS*

Notes: Only the mentees who worked as nurses or ANMs were included in this analysis

^a^Baseline scores were measured during the first mentoring session (Sept to Nov 2016)

^b^End scores were measured during the last mentoring session (Jan to Mar 2018)

^c^PPH: Post-partum haemorrhage

^d^Assessed at the study mid-point and end

^e^Assessed in all CEONCs and in some BEONCs only (those with equipment available)

### Mentoring effectiveness on knowledge

The proportion of mentees who obtained a good knowledge assessment score increased to 86.1% at the third mentoring session, indicating a significant and positive increase of 28.3% in knowledge (*p* < 0.001) (Table 2). The mentees who gained the most knowledge were those working in birthing centers (+ 52.0%), those not trained as SBAs (+ 36.1%, p < 0.001), those with the lowest level of nursing education (+ 30.3%), and those working as ANMs (+ 28.8%).

### Mentoring effectiveness on clinical performance

The overall clinical performance increased at the end of the program (+ 62.7%, p < 0.001). The greatest improvement was observed in the birthing centers (+ 80.7%) among SBAs (+ 72.3%), ANMs (+ 67.6%) and staff with the lowest level of nursing education (+ 67.3%).

During the last mentoring session, significant improvements were observed for all skills assessed (Table 3). The skills with the greatest improvements were the management of haemodynamic shock (+ 57.2% of mentees performed well compared with those at baseline), management of eclampsia (+ 56.9%), new-born resuscitation (+ 56.7%) and completion of a normal delivery (+ 55.0%). The skills that improved the least (decontaminating instruments and putting on sterile gloves) were those that were already mastered at baseline. Comparisons of changes in clinical assessment scores did not show any significant differences between SBAs and non-SBAs (Additional file 3: Table S1). This result means that although this mentoring program used tools that were initially designed for SBAs, both SBAs and non-SBAs improved in their knowledge and ability to demonstrate clinical skills. Similar comparisons between ANMs and staff nurses showed that ANMs’ gains in clinical performance were higher than those of nurses for the two following skills: managing shock due to post-partum haemorrhage and conducting a normal delivery (+ 30.0% among ANMs vs +  21.0% among nurses, *p* = 0.005; + 21.0% among ANMs vs +  18% among nurses, *p* = 0.03, respectively) (Additional file 4: Table S2).

### Mentoring effectiveness at the individual nurse level

The improvement of knowledge and clinical skills was also confirmed at the individual level (Table 4). Of the 172 mentees who underwent at least two knowledge assessments, 75.0% increased their scores. A significant positive increase was also found for all clinical skills assessed. More than 90% of mentees who were assessed at least twice improved their scores for the following skills: completing a normal delivery, managing post-partum haemodynamic shock, resuscitating a new-born, and managing eclampsia. The two skills which least improved were those best performed at baseline (using sterile gloves: 91.4% of mentees obtained a score > 85% at baseline; decontaminating instruments: 79.6% with high scores at baseline). In most cases, when the scores were lower at the end of the program, the drops in performance were minimal. For instance, the mean drop for the seven staff getting lower scores for managing an eclampsia case was − 6%.

**Table 4:** Individual changes in assessment scores from baseline to the end of the mentoring program in Nepal from 2016 to 2018

	Mentees assessed	Score change at the end of the program						Overall test significance^a^
		*Mentees with a higher score*		*Mentees with a lower score*		*Mentees with no change*		
	No.	n	%	n	%	n	%	*P*
Knowledge assessment	172	129	75.0	13	7.6	30	17.4	*< 0.001*
Skills assessments								
Completing a normal delivery	166	153	92.2	3	1.8	10	6.0	*< 0.001*
Managing shock due to post-partum haemorrhage	142	130	91.5	4	2.8	8	5.6	*< 0.001*
Resuscitating a new-born	164	149	90.8	4	2.4	11	6.7	*< 0.001*
Managing an eclampsia case	145	131	90.3	7	4.8	7	4.8	*< 0.001*
Interpreting a partograph	171	150	87.7	7	4.1	14	8.2	*< 0.001*
Safely referring a woman or a new-born	139	119	85.6	8	5.8	12	8.6	*< 0.001*
Promoting kangaroo mother care	138	118	85.5	4	2.9	16	11.6	*< 0.001*
Using sterile gloves	143	36	25.2	1	0.7	106	74.1	*< 0.001*
Performing condom tamponade	99	66	66.7	7	7.1	26	26.3	*< 0.001*
Decontaminating used medical equipment	125	56	44.8	3	2.4	66	52.8	*< 0.001*
Performing manual aspiration^b^	43	35	81.4	4	9.3	4	9.3	*< 0.001*
Performing a vacuum delivery^b^	58	46	79.3	2	3.4	10	17.2	*< 0.001*

Score change was computed for each mentee as the difference between the first and last assessments. A higher score at the end indicated that the mentee improved her knowledge or clinical skill

^a^Significance was assessed using the Wilcoxon matched-pair signed-rank test; P was estimated for two-sided test, testing for both positive and negative increases at the end of the programme. The test showed that none of the loss in performance score was statistically significant

^b^For skilled birth attendants only

## Discussion

### Main findings

Our study showed that prior to the mentoring program, the nursing teams in charge of deliveries in CEONCs, BEONCs and birthing centers of Nepal demonstrated limited ability to perform several life-saving procedures in emergency obstetrical and neonatal care, regardless of their initial training. In particular, these teams were poorly able to manage shock due to post-partum haemorrhage or an eclampsia crisis. Additionally, common procedures, such as completing a normal delivery according to the national standards or correctly interpreting a partograph and taking action, were not mastered by the maternity staff in charge of deliveries. These results may be explained by the limited clinical experience and practice exposure of these young nurses, as illustrated by the fact that nurses working in CEONCs (with a higher caseload) had better knowledge and skills than those of other staff. Nevertheless, this result raised questions about the quality of their pre-service training. These findings corroborate a recent study that concluded the presence of “limited capacities of maternity staff in Nepal, despite important investments from the Government” [25]. However, we found that ANMs and nurses trained as SBAs had better knowledge and better overall clinical scores than their non-trained peers, which shows the impact of the efforts by the MOH since the mid-2000s to upgrade its active workforce in maternity settings.

Notable improvement was observed after this structured mentoring program was provided by senior nurse peers. The mentees significantly improved their knowledge in MNC, with 86.1% of them reaching a ‘good level of knowledge’, as defined by the MoHP of Nepal. The mentees also improved their ability to perform the 12 core MNC clinical skills of this program, with marked improvements in the following three procedures that save mothers’ and new-borns’ lives: managing eclampsia, managing post-partum haemorrhage shock, and resuscitating a new-born.

This mentoring program appeared to have greatest impact among the staff with the lowest level of nursing education and in the lowest level of health facilities providing maternity care, namely, the birthing centers, where most of the staff are ANMs who work in isolated and remote areas despite their limited education. Interestingly, the mentoring program had a strong positive effect on non-SBAs, although the QI tools used were primarily designed for SBAs. We strongly believe that this is due to the individual-to-individual approach used in the programme, which allowed mentors to tailor their input according to the needs of each mentee.

### Limitations of the study

The lack of a control group means that we are unable to determine to what degree the changes in outcome are solely the result of the intervention. Moreover, we assessed the nurses’ clinical skills mostly using case scenarios and simulation models due to the limited caseload in some health facilities, which do not replace clinical observations during the provision of care to actual patients. This difficulty is inherent to QI programs in EmONC. Emergencies in maternity settings are often unpredictable and rare in places with low caseloads [26]. Moreover, the data we collected did not allow us to identify the potential impact of the program on maternal and new-born outcomes. Another limitation of this study is that the validity and the reliability of the knowledge and skills assessments are not known.

### Sustainability

The institutionalization of this mentoring program within the MoHP in a sustainable form was initiated as early as the beginning of the program by the FHD/MoHP and the National Training Health Center. More than 90 government mentors were already trained and certified by the FHD/MoHP and the National Health Training Center at the end of the program. Funds have been secured by the MoHP to cover their travel and accommodation costs, as well as to fund extra staff to fill the gaps created while mentors are away from their post to perform their mentoring activities.

However, the overall sustainability of the program remains unclear, mainly due to the local political context, as the health system is undergoing a complete restructuring to fit into a newly implemented federal system. The program team suggested that the MoHP explore the feasibility of developing ‘cascade-mentoring’, with local mentors further trained and mentored by provincial mentors, as successfully implemented in Uganda in the area of MNC [26]. Nepal has, however, taken a very promising step forward by launching a midwifery education program [27–29]. It is expected that the soon-to be deployed midwives will help improve the clinical skills of ANMs and nurses.

### Lessons learned

Given the geo-economical context of Nepal and its current transition from a central government to a regional system, it will take time before all birthing centers have a trained midwife or SBA-registered nurse. Meanwhile, and because SBAs are better equipped to address normal deliveries and obstetrical complications than their non-trained peers, SBA training is still provided and should be scaled-up and accelerated for all employed maternity staff and combined with regular refresher courses and supervision/mentoring by experienced nurses. Plans are currently being made by the MoHP to reshape the existing SBA course into a ‘bridging course’ to become a certified 1-year midwifery course for all staff who have previously completed the 3-year PCL nursing course. Trained staff will be registered as midwives in the Nepal Nursing Council.

Among the lessons learned from this program, we found that on-site clinical mentoring in remote locations requires motivated mentors, engaged mentees and support from hospitals and health facility management committees. During qualitative focus group discussions held during the closing project meeting, the program team, including the mentors, also observed that mentoring builds teamwork, staff confidence, motivation, and a sense of ownership and pride. This finding may be due to one innovative feature of this mentoring program: the bundle approach linking clinical and management mentoring. Given that high-quality clinical services strongly depend on management support for the provision of basic infrastructure, water, power, equipment and supplies, as well as regular cleaning and maintenance, all clinical mentoring activities were organized jointly with the management mentors, promoting synergy, interaction and collaboration between the clinical and management teams, as seen elsewhere [26]. At the facility level, clinical and management teams developed the habit of discussing context-specific needs and of developing and effectively implementing gap-closing plans.

Among the factors that adversely affected the implementation of the program, in addition to the low caseload mentioned in the limitations section, we note the difficulty of maintaining the cohort of nurse-mentees due to frequent staff transfers and vacancies (mainly training opportunities or pregnancy leaves). An additional challenge was the changing political context, as Nepal’s constitution was revised during the program, and the MoHP moved from a centralized ministry to a federal context.

## Conclusion

Quality of care has recently gained much attention, as it is now clear that universal health coverage will not be reached without improvement in the quality of health services [30]. Despite its limitations, our study adds to the body of evidence on methods of quality care improvement. This on-site clinical mentoring program, coupled with health facility management mentoring, effectively contributed to increasing clinical competences and performance of health workers in maternity settings. Still the study did not measure the programme’s impact on patient safety. This program functioned as an integral part of the MNC program of the MoHP through the use of tools developed by the National Health Training Center. Nevertheless, the sustainability of this program is being challenged by the current restructuring of the overall health system toward a federal government. However, this health system restructuring offers an unprecedented window of opportunity that should be utilized to strengthen human resources for MNC. One-site mentoring could be a good option, as commonly implemented in GIZ-supported programs, to help change clinical practices at the frontline to deliver high-quality healthcare “at the right time, in the right place, by the right care provider, while minimizing harm and resource waste and leaving no one behind” [31].

## Supplementary information

**Additional file 1.** Quality domains targeted by health facility management mentoring.

**Additional file 2.** Content of the knowledge assessment questionnaire.

**Additional file 3: Table S1.** Changes in assessment scores from baseline to end line by SBA status, Nepal, 2016-2018.

**Additional file 4: Table S2.** Changes in assessment scores from baseline to end line by mentees' function status, Nepal, 2016-2018.

## Data Availability

The datasets used and/or analysed during the current study are available from the corresponding author on reasonable request.

## Abbreviations

BEONC: Basic EmONC center
CEONC: Comprehensive EmONC center
EmONC: Emergency obstetrical and new-born care
FHD: Family Health Division
GIZ: Deutsche Gesellschaft fur International Zusammernarbeit
MMR: Maternal mortality ratio
MNC: Maternal and new-born care
MoHP: Ministry of Health and Population
NMR: Neonatal mortality rate
QI: Quality improvement
SBAs: Skilled birth attendants
