# Supplementary material for: On-site clinical mentoring as a maternal and new-born care quality improvement method: evidence from a nurse cohort study in Nepal
Source: BMC Nurs. 2020 Jan 8;19:3. doi: 10.1186/s12912-019-0396-1 (PMC6950904; doi:10.1186/s12912-019-0396-1)
Supplement: Supplementary file 1 — Additional file 1. Quality domains targeted by health facility management mentoring. [file 12912_2019_396_MOESM1_ESM.docx]

**Additional file 1: Quality domains targeted by health facility management mentoring**

| **Quality domains** | **Details** |
| --- | --- |
| 1. Health facility management | including the availability of delivery services, of monthly maternal and perinatal reviews |
| 1. Infrastructure | including the availability of electricity and water |
| 1. Patient dignity | including cleanliness of the facility, availability of screens and curtains between delivery table |
| 1. Staffing | including availability of SBA trained staff, of doctors trained and anesthesia to perform c-sections |
| 1. Supplies and equipment | checking the availability of essential supplies a for obstetrical and newborn care |
| 1. Supplies of drugs | checking the availability of drugs for obstetrical and newborn care |
| 1. Clinical practices | including the availability of various guidelines and protocols, the verification of completeness of recent partographs and facility register |
| 1. Infection prevention | including the availability and use of sterilization equipment, waste management procedures |
