# Supplementary material for: On-site clinical mentoring as a maternal and new-born care quality improvement method: evidence from a nurse cohort study in Nepal
Source: BMC Nurs. 2020 Jan 8;19:3. doi: 10.1186/s12912-019-0396-1 (PMC6950904; doi:10.1186/s12912-019-0396-1)
Supplement: Supplementary file 2 — Additional file 2. Content of the knowledge assessment questionnaire. [file 12912_2019_396_MOESM2_ESM.docx]

**Additional File 2: Content of the knowledge assessment questionnaire -**

This questionnaire was extracted from the national Follow up and Enhancement Programme (FEP) manual for Skilled Birth Attendants, Nepal

| Topic | Question number | Content |
| --- | --- | --- |
| Antenatal care | 1 | Goals of basic MNC |
|  | 2 | Basic component of MNC |
| Partograph | 3 | Parameters that need to be filled in a partograph |
|  | 4 | Cervical dilatation plotting during active phase of labor |
|  | 5 | Measures to take for unsatisfactory progress of labor |
|  | 6 | Signs of fetal distress during labor |
|  | 7 | Conditions leading to birth asphyxia |
|  | 8 | Measure to take in case of fetal distress |
| Normal delivery | 9 | Appropriate order of steps in active management of third stage of labor |
| Vacuum delivery | 10 | Conditions for vacuum delivery |
|  | 11 | Description of posterior fontanel |
| Complicated procedure | 12 | Assessment of woman with vaginal bleeding after 28 weeks of pregnancy |
|  | 13 | Definition of abruption placenta |
|  | 14 | Reasons for vaginal bleeding after birth and well contracted uterus |
| Newborn | 15 | Use of oxygen during resuscitation |
| Eclampsia | 16 | Associated signs and symptoms during pregnancy induced hypertension |
|  | 17 | Surveillance of a woman after administration of magnesium sulfate |
|  | 18 | Loading dose of magnesium sulfate |
|  | 19 | Signs and symptoms of postpartum depression |
| Infection prevention | 20 | How to wash hands when water is not available |
|  | 21 | Aim of decontamination |
|  | 22 | Method of decontamination of instruments |
|  | 23 | Method of high level disinfection |
|  | 24 | Management of harmful waste from health facility |
|  | 25 | Role of a SBA midwife, nurse or doctor |
|  | 26 | Universal precautions to follow while treating patients |
